# Global Collaborative Social Network (Share4Rare) to Promote Citizen Science in Rare Disease Research: Platform Development Study

**DOI:** 10.2196/22695

**Published:** 2021-03-29

**Authors:** Roxana Radu, Sara Hernández-Ortega, Oriol Borrega, Avril Palmeri, Dimitrios Athanasiou, Nicholas Brooke, Inma Chapí, Anaïs Le Corvec, Michela Guglieri, Alexandre Perera-Lluna, Jon Garrido-Aguirre, Bettina Ryll, Begonya Nafria Escalera

**Affiliations:** 1 The Synergist Brussels Belgium; 2 Sant Joan de Déu Research Institute Esplugues de Llobregat Spain; 3 Omada Interactiva SL Barcelona Spain; 4 John Walton Muscular Dystrophy Research Centre Newcastle University Newcastle United Kingdom; 5 Stichting United Parent Projects Muscular Dystrophy World Duchenne Organization Amsterdam Netherlands; 6 ClicLab Barcelona Spain; 7 Center for Biomedical Engineering Research Universitat Politècnica de Catalunya Barcelona Spain; 8 Melanoma Patient Network Europe Uppsala Sweden

**Keywords:** Share4Rare, rare disease, citizen science, participatory medicine, natural history, genotype, phenotype

## Abstract

**Background:**

Rare disease communities are spread around the globe and segmented by their condition. Little research has been performed on the majority of rare diseases. Most patients who are affected by a rare disease have no research on their condition because of a lack of knowledge due to absence of common groups in the research community.

**Objective:**

We aimed to develop a safe and secure community of rare disease patients, without geographic or language barriers, to promote research.

**Methods:**

Cocreation design methodology was applied to build Share4Rare, with consultation and input through workshops from a variety of stakeholders (patients, caregivers, clinicians, and researchers).

**Results:**

The workshops allowed us to develop a layered version of the platform based on educating patients and caregivers with publicly accessible information, a secure community for the patients and caregivers, and a research section with the purpose of collecting patient information for analysis, which was the core and final value of the platform.

**Conclusions:**

Rare disease research requires global collaboration in which patients and caregivers have key roles. Collective intelligence methods implemented in digital platforms reduce geographic and language boundaries and involve patients in a unique and universal project. Their contributions are essential to increase the amount of scientific knowledge that experts have on rare diseases. Share4Rare has been designed as a global platform to facilitate the donation of clinical information to foster research that matters to patients with rare conditions. The codesign methods with patients have been essential to create a patient-centric design.

## Introduction

Rare diseases are characterized by their low prevalences, and often, for being chronic, debilitating, or life-threatening conditions [1]. Despite this, over 400 million people worldwide are estimated to be affected by a rare disease [2]. In Europe, a disease is defined as rare when it affects less than 1 in 2000 citizens, accounting for an estimated 30 million people in the European Union [3]. Between 6000 and 8000 different rare conditions have been identified to date [4].

Most rare diseases (80%) have a genetic origin, and between 50% and 75% have an onset at birth or during the first years of life [5], with a wide variety of symptoms and signs that vary, not only from disease to disease, but also from patient to patient. Indeed, relatively common symptoms can hide underlying rare diseases, leading to misdiagnosis. It is estimated that approximately 25% of patients with a rare disease wait between 5 and 30 years to obtain a diagnosis (if they ever obtain one), and it is estimated that, during that time, 40% receive an incorrect diagnosis [6]. This heterogeneity in diseases and symptoms, along with geographic dispersion, makes it more difficult to access reliable information (if it exists) or to gather a significant number of patients for research to better understand the cause of each condition. It is also more difficult to describe the natural history of the disease in order to facilitate patient care and treatment. Furthermore, numerous countries do not have mandatory registries of patients, making it almost impossible to know the epidemiology of the diseases and the incidence in their populations.

From a medical point of view, ultrarare diseases, also called *orphan diseases*, often do not have any treatment options. This leads to use of off-label drugs without scientific evidence for 73% of cases, and wasteful or harmful treatment may occur [7]. By providing professionals with information about frequency, signs, symptoms, age of onset, and disease progression, potential outcome measures can be identified for later therapeutic trials. Once the variability and rate of progression of a specific disease-related sign or symptom are known, the information can be utilized as a control in the design of a clinical trial. The natural history description of a disease helps clinicians with early diagnosis and aids in the counseling of patients and families.

The internet has increased public access to health information and transformed patient behaviors. New services are offered in the health field based on the power of the interconnection of this network. For example, an overwhelming majority of parents (89%) accessed the internet before meeting with genetic providers at metabolic treatment centers [8]. Another interesting statistic shows that 18% of users connected to others with their disease through the internet [9]. However, few clinicians recommend websites to parents at the time of diagnosis as there are no trusted sources of information available for rare disease patients and caregivers or virtual meeting spaces.

Participatory health is a growing area, with individuals using health social networks, crowdsourced studies [10], smartphone health apps, and personal health records to organize their own research studies through health collaboration communities created especially for the purpose of self-experimentation and the investigation of health-related concerns.

In this landscape, Share4Rare is a project supported by the European Commission through the Horizon 2020 program (GA780262). The project aims to (1) develop an online platform to collect information about patients affected by rare diseases in order to build a global community, (2) connect people and patient organizations and communities, and (3) promote research by collecting clinical information to describe the natural history of rare diseases.

The aim of this paper is to describe the methodology that was followed in the Share4Rare project to build a collaborative social network with the 3 previous objectives—educate, connect, and foster research based on the donation of medical information from the users of the platform. To cocreate the platform, several co-design sessions were organized with the main stakeholders: patients, patient representatives, clinicians, and researchers.

Altogether, the methods and analysis of the sessions were used to build our platform based on (1) including a patient-centered perspective in the design and development of research projects about rare diseases through clinical information donation, (2) promoting a supportive collaborative social network to enhance synergies between families so they can interact and improve disease management, (3) connecting patients to their respective patient organizations to grow and empower all rare disease communities, (4) generating a sense of community to help reduce the isolation of and stress levels in families living with a rare disease and to improve their quality of life, and (5) providing a virtual space for patients and caregivers to connect and learn from each other as well as from health care professionals.

## Methods

### Cocreation Process

**Figure 1:**
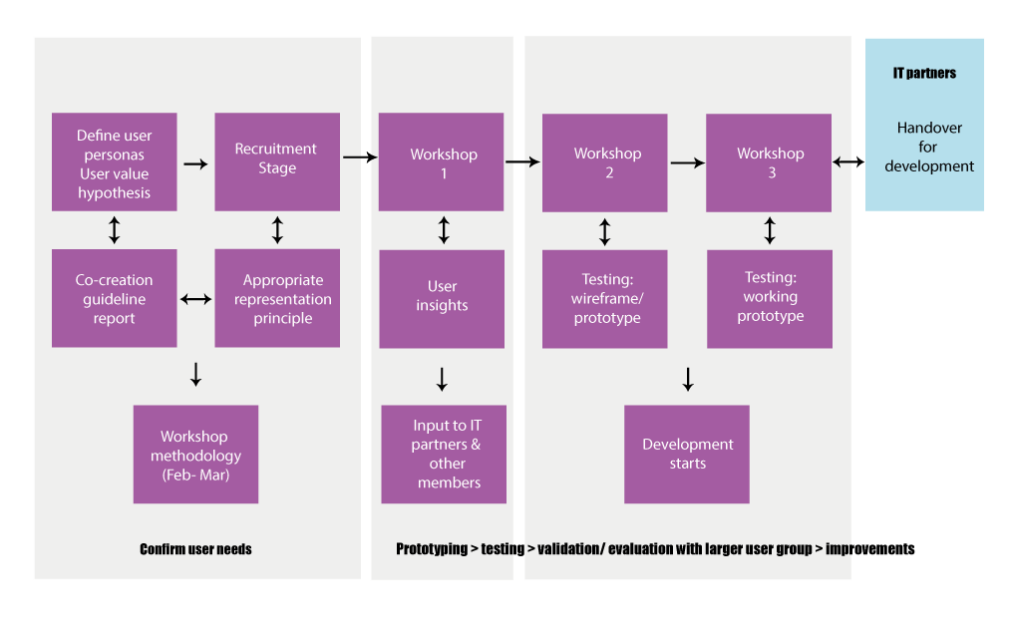
The diagram illustrates the key steps in the cocreation process, highlighting our iterative approach to the design of the platform.

As a participatory platform centered around the needs of patients and caregivers affected by rare diseases, Share4Rare integrated various stakeholders’ perspectives in the initial design of the platform through a cocreation process. The adoption of cocreation frameworks (Figure 1) aimed to ensure that end users were able to directly influence how the platform would take shape, and thus, facilitate their joint ownership of the end product.

Stakeholders, whose input was critical to the delivery of a useful platform and representing the target audience who could benefit from the platform, were invited to participate. The target audience included specific patients and patient groups along with the caregivers of minors, as well as clinicians and researchers. The inclusion criteria specified that participants needed to be able to travel to the workshop locations or participate via video or teleconference and be able to understand English or Spanish.

The recruitment process began with creating user profiles for approval by the organizations involved in the project. Then, an invitation was created along with an engagement plan. The plan was reviewed along with the target of users represented in different workshops. The consortium of organizations involved in the project reached out to our networks (other organizations with which we collaborate) to suggest participants and to organize all the practicalities and accessibility considerations if any were needed.

### Theoretical Framework

**Figure 2:**
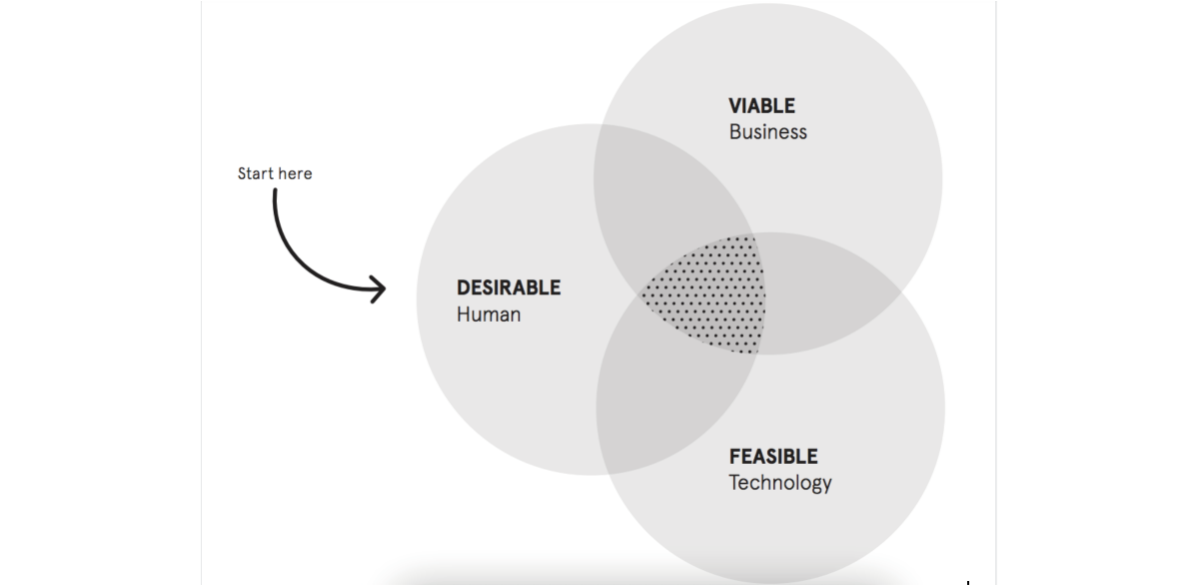
Human Centered Design model from [9].

Theoretical frameworks were used to guide the design process. The Human Centered Design approach by IDEO [11] was adopted as a framework to guide the cocreation workshops (Figure 2). This approach aims to ensure an outcome that balances meeting users’ desires, offering a viable business model and technical feasibility.

It provides a dedicated space and facilitation for cocreation activities while ensuring a balanced and diverse representation of stakeholders. It is broken down into 3 phases: Inspiration, Ideation, and Implementation:

The *Inspiration* phase is an information-gathering phase aimed at understanding users. Observing users’ everyday lives and challenges provides a deep understanding of their needs. Research and analysis of other sources form a foundation to build upon.

The *Ideation* phase assesses the observations gathered in the Inspiration phase to identify possible solutions that fit with business or project needs, user desires and technical feasibilities. Prototypes are developed, shared then iterated based on the feedback received.

Finally, the cocreated prototypes are built during the *Implementation* phase; for Share4Rare, this refers to the development of the platform. Again, an iterative process allowed for multiple rounds of feedback.

### Preworkshop Phase

Prior to the workshops, information was used within the initial project proposal as the basis for a collaborative document describing the *personas* of the expected primary users of the platform and how these users might behave on the platform, based on their individual needs.

To validate and improve this information, an online survey was used to gather feedback initially from patient advocacy groups closest to patients and caregivers, and later, from the rest of the consortium. Members were asked to check, prioritize, and complete the user personas, user scenarios, and feature types identified. The outcomes from this first step helped us decide the activities and group interview questions for Workshop 1.

### Cocreation Workshops

The workshops were structured in a successive order following the Human Centered Design approach [11]. Workshop 1, the first cocreation workshop, focused on the Inspiration phase. It aimed to understand the needs of patients, caregivers, and clinicians to ensure that the platform was designed with their needs in mind. Workshop 2, the second cocreation workshop, focused on the Ideation and Implementation phases, validating the initial conclusions drawn in Workshop 1. Workshop 3, the third cocreation workshop, focused on testing the hypotheses for the unique value proposition to enable us to confirm the main features and direction the platform development should take.

Patients and caregivers (sample size target: 10 to 12) were included in each of the 3 cocreation workshops. We specifically looked for patients without a diagnosis, newly diagnosed patients, long-term care patients, and palliative care patients. Caregivers attended the workshops on behalf of pediatric patients. Patient advocates were involved in the selection of participants, and we had more than 3 European countries represented in every workshop.

During these workshops, we employed a simplified Delphi methodology [12], which helped build consensus while ensuring all participants had an equal voice in decisions. Following the Human Centered Design [11] ensured that all participants had opportunities to contribute throughout the design process. This allowed end users to directly influence how the platform would take shape, ensuring joint ownership of the end product.

## Results

### Workshop 1: Methodology and Conclusions

The first session, with patients and caregivers, began with group interviews. The group was divided into smaller, diverse groups of caregivers and patients who, supported by a moderator, were asked to answer a series of questions to understand their needs.

This provided key information on main hurdles and challenges the possible users are facing in their day to day life, habits regarding the usage of digital tools, and some generic expectations from a platform similar to Share4Rare (Textbox 1).

The second session, with clinicians and researchers, also began with group interviews. Participants were asked a series of questions to understand their specific needs in the Share4Rare community. The clinicians and researchers were then asked to prioritize needs previously identified in the preworkshop phase.

In the second part of the patient and caregiver session, a game helped us to prioritize user needs previously identified in the preworkshop phase among the focus group. Each participant was given an equal amount of false money and presented with several boxes representing banks and labeled with a need type. They were asked to distribute their money according to how important they considered that need. At the end of the exercise, the amount of money in each bank was counted. A group discussion explored why each need was or was not chosen.

When prioritizing some proposed features, the patients and caregivers clearly preferred having the ability to contribute to research, closely followed by connecting with similar people to them, as well as finding information about the diseases (Table 1).

The first iteration of the Share4Rare platform design and interaction model was drafted after both sessions.

The most needed features or feature types (Table 2) required further development and prototyping to be tested with a wider group of users.

**Insights from Workshop 1 with different stakeholders.**

**Patients and caregivers**

- Almost all patients or caregivers use the internet to reach out to other people in the same situation
- Patients or caregivers would like an easy way to interact; notifications are also important to keep them up to date
- Everyone needs a space where they can find various levels of information on the disease; the language used needs to be appropriate
- It would be interesting to have information about symptoms and what they could mean (there are a lot of patients without diagnoses)
- There needs to be a space for open dialogue between patients and doctors
- Parents/patients would like to have some kind of job board adapted to their needs

**Clinicians and researchers**

- Ability to connect with similar experts in the field
- An exercise to map global research projects in rare diseases and identify gaps would greatly help their work
- The primary caregiver needs to be educated on rare diseases and how to refer patients to bigger diagnostic centers
- Integration with European Reference Networks is essential
- Collaborations with other institutions seem to be a main issue for the clinicians
- Having access to the latest news in the field
- Essential to have a section to help educate general public, patient associations, funders on the procedures of the research process, main needs, etc
- Tackle lack of funding in some way through the platform

**Table 1:** Features prioritized after Workshop 1.

Feature	Score
Contribute to research efforts	15
Connect with other people	10
Find information	6
Access latest news	5
Mental health support	4
Get support during the diagnostic journey	3
Connect with my doctor or medical facility	2
Read about other people like me	1

**Table 2:** Summary of the most needed features or feature types identified following Workshop 1.

Feature		Needs
**Level 1 (medical, open information)**		
	Wiki space—easy to read medical information (easily grouped by types of conditions, diseases, pediatric or not; information to be constantly kept up to date); important—information on genetic risk and prevention measures	Easy to read, in lay language Easy to find relevant topics Easy to add or modify by dedicated professionals (not by community) Should link to external resources Consider “verified” button or something indicating quality
	Dedicated section to diagnosis (here we can have a grouping on symptoms as the patients suggested)	Same as above
	List of specialized centers	An easy to navigate map Should have a way to visualize simple details for each center
**Level 2 (communities)**		
	Support groups on various topics (Based on the content interaction model: patient/caregiver can decide on topics he/she is interested in)	Very easy to quickly find relevant topics or discussions Have tags or other system to label conversations with more than one keyword to make them easier to search for Browsing feature or conversation feed for those who do not have a particular question but just want to see what is there Easy to join a discussion Quality of the conversation is important Easy to navigate to direct messaging if they want to make the conversation private
	Direct messaging option	Safe, private, easy to use Easy to connect with the forum
	Maps with various specialists to help parents (mental health specialists, lawyers, social workers, etc)	An easy to navigate map Ideally, we should have a “sign up to help” call to action for the professionals mentioned above
	A method to connect with expert clinicians	Ability to view lists with vetted clinicians that could answer the needs of the various users. The platform should provide the first point of contact and should not replace in-person clinical assessments
**Level 3 (medical data)**		
	Search engine (transversal) for clinical trials, drugs, therapeutic methods	Intuitive way to search for various subjects (new/ongoing clinical trials, disease based, etc)

### Workshop 2: Methodology and Conclusions

The workshop began with a *gallery walk exercise* in which participants were presented a gallery of Share4Rare platform screenshots and features. They were then invited to ask questions and give their first impressions and feedback. A guided presentation of the Share4Rare platform prototype followed with a group discussion on each aspect was presented. Targeted questions explored the points raised by participants during the gallery walk in greater depth.

The group gave feedback on various specific features proposed by the information technology partner. For the storytelling feature, participants prioritized the idea of filtering stories by disease type and role, as well as the ability to add links to owned materials & resources. A key question that was raised was “how are children protected on the platform?” Parents could be happy to share lots of information, but as their child grows, they might be less happy having the information visible.

Regarding the question and answer feature, participants discussed how users should be listed in relation to others (based on interest, active topics, etc), as well as the ability to add or remove topics of interest. For the participants, it was important to note how we would manage the answers since some patients or caregivers may give answers as if they were doctors or experts that could be inaccurate or misleading. In smaller communities (such as Facebook groups), administrators step in and manage the situation. The participants also decided that only patients and caregivers should be allowed in the forum. Suggestions for content were also made, especially around the quality of life topic and language concerning children.

Additional conclusions from the group further helped guide platform design. For example, the group was against a popularity ranking in the platform and raised various questions around privacy, especially when it came to the option of navigating as an anonymous avatar (since the topics of interest can still serve as an identifier).

The undiagnosed group raised the point that it can be difficult to know where to start when navigating the platform. Users also required the ability to use filters or navigate based on disease types and symptoms and to implement the ranking of topics or content pieces based on an algorithm that takes into account topics the user has mentioned they might be interested in and navigation habits.

After the conclusion of the second cocreation workshop, participants felt that the unique value proposition of the platform was not strong enough and that there was a risk that Share4Rare would not succeed in attracting enough users.

As a result, participants decided to modify the methodology for the third workshop. The platform design was revised to articulate the platform features and unique value proposition more clearly.

### Workshop 3: Methodology and Conclusions

The final cocreation workshop focused on the unique values of the Share4Rare platform and the specific feature needs of patients and caregivers. Participants were asked about their perceptions, opinions, beliefs, and attitudes regarding the Share4Rare platform prototype. A full day of interviews, role-playing creativity games, and design challenges helped to determine the subsequent development steps: (1) Welcoming activities introduced participants and facilitators. (2) Participants interacted with and tested the prototype Share4Rare platform, allowing us to identify areas for revision and validate its unique selling point. (3) Predetermined questions were asked to encourage participants to share their opinions and ideas as well as listen to and engage with the opinions and ideas of others in a small and safe group setting. (4) A cocreation activity, in which participants built their own version of the Share4Rare platform, brought participants into the design process by rapidly prototyping their own solutions to the problems. (5) Clinicians and researchers received targeted surveys aimed at validating the platform’s development and unique value proposition.

The workshop provided some key information that helped prioritize key aspects—from feature creation to the value proposition and event content creation.

We realized that it was important to deliver the right information at the right time to the user, as a patient or caregiver may not feel comfortable getting in contact with people or receiving information from a later stage of the disease. That is why the key aspect was the ability to find other people with similar problems, someone to talk to or to help them understand the condition and procedures. Users wanted to know about the cause and the progression of the disease when there is no treatment. Each disease is different; therefore, they would like to see specific filters for each disease and other filters such as age or level of clinical understanding. Anonymous navigation—the ability to “watch from a distance”—was mentioned many times. The future users mentioned many times that they need to know what is located close to them in terms of facilities and expertise (keeping in mind that there are different types of experts, such as patient experts). We also understood from the discussions that we would need different levels of access to allow the users decide how much they share and how much they interact with other people. Content-wise, patients and caregivers mentioned the need to discuss comorbidities, mental health, physical therapy, palliative care, and other general topics.

The patients and caregivers were given a survey and asked to prioritize various content topics and subtopics previously agreed upon by the consortium. Keeping in mind that this type of feedback is usually biased as users prioritize according to their needs, we reorganized the topic and subtopics to reflect prioritization (Textbox 2).

The participants also had the opportunity to add topics that they believed were missing, which included symptom control; database with various specialists (neurology, orthopedics, cardiology, respiratory); standards of care; advocacy; legal issues in trials; end-of-life care—advanced care planning (resuscitations, wills, funeral planning), power of attorney, and capacity; independent living, equipment, and professional caregivers; complementary nutrition (eg, supplements); disability models; and existing tools (for example, integration with Orphanet, European Reference Networks, and local tools).

**Topic prioritization.**

1. Understand your disease

- Find your rare disease
- Reference centers
- Rare diseases in facts
- Undiagnosed diseases
- Genetic counseling
- Genetics
- Basic research

2. Treatments

- Clinical trials
- Medicines
- Psychology
- Physiotherapy
- Orthopedics
- Nutrition
- Palliative care
- Speech therapy

3. Quality of life

- Emotional support
- Education
- Patient associations
- Caregivers
- Accessibility and disability issues
- Leisure and sport

4. Legal issues

- Grants and subsidies
- Ethics
- Regulations

### Cocreation Beyond the Workshops

The workshops provided significant and meaningful insights into the needs and requirements of the users, but further collaborative work was needed with the consortium. First, to advance the discussions around the unique value proposition of the platform, and second, to prioritize the extensive feature list which came out of the user cocreation activities.

Consortium members identified and ranked the primary value propositions of the platform in an online survey based on the outcomes of the previous cocreation activities (Textbox 3). A decision-making matrix assisted the consortium in debating the platform’s final development plan (Table 3).

**Value proposition survey results, answering the question “how will we distinguish ourselves from other solutions so people actually use Share4Rare?”**

**Unique value proposition**

1. One network showing the 360-degree picture, not just the medical perspective
2. Get targeted, quality information faster and using fresh, innovative delivery from peers and/or expert people
3. Capture common pathways/experiences to gain time and better management of disease
4. Give an active role to patient organizations to support their members
5. Unique connection point between patients and health care professionals
6. Provide practical support to health care professionals across rare diseases, including access to medical information generated by expert doctors and updated with real data from patients.
7. Easier for patients and caregivers to access or request information that can allow a second opinion

**Table 3:** Top features and feature types—which features will make the unique value proposition a reality?

Rank	Feature type	Top ranked features (top 50% within each type)
1	Connect patients, caregivers and clinicians through data	1. Donate clinical data to facilitate the generation of new and qualified knowledge about the disease 2. Check information that can facilitate the access to a second opinion using clinical data
2	Providing education and support	1. Content reviewed by an editorial board/ volunteering community management team will receive a “trusted source” stamp. All other content will still appear in the platform but with a quality warning (eg, “This information has not been verified yet”) 2. Filter the search per disease type 3. Access medical chapters—books with relevant information about diseases 4. Search engine across all content types (medical information, forums, questions, etc) 5. Upload information and materials on diseases 6. Search for a symptom 7. Filter by language 8. Filter per disease stage (certain diseases only)
3	Find a mentor who can support the experience with the disease	1. Find a local disease ambassador, patient advocate or mentor 2. Ask for specific information that can worry them and that can be provided or curated by the mentor 3. Mentors have a dashboard to easily manage mentorship tasks
4	Community support	1. Forum feature 2. Private forums, only accessible by invitation 3. User can follow tags and forum threads, appearing on the user’s dashboard 4. Allow users to contact through private messaging inside the platform
5	Resources for disease management & support	1. Navigate a world map & find health care facilities (diagnostic centers, treatment centers, etc) 2. Navigate a world map to find various experts. Expertise filters would help navigate the map 3. Find other users by using profile filters (caregiver, patient, doctors...), interest or geographic filters
6	Interaction with a health care professional	1. Health care professionals will be able to share their expertise with other clinicians that can have patients from the two pilot groups of conditions 2. Health care professionals will be able to sign up in the platform and volunteer to support the rare disease community

## Discussion

### Analysis of the Co-design Findings

After these analyses and cocreation workshops were completed, a proposal of the platform was developed: (1) Education layer: Existing curated content will be linked to the platform. At the same time, for diseases where a minimum of information is not fully covered, new curated material will be developed in order to reach all of the diseases included in the pilot of the project. (2) Share layer: A sense of community will be created by a network of interactions that will allow patients to connect to peers or clinicians based on the answers they give through different questionnaires. These data will be collected, analyzed through machine learning methods and the network around the user will be updated daily, allowing him or her to adjust and find the correct person to contact in each case. (3) Research layer: This is the gold value of the platform. Data donation will be accomplished through several questionnaires. In the case of adult patients or relatives (legal guardians) of a pediatric patient in the pilot disease areas, the number of questionnaires will be aligned with the complexity of the disorder.

All of this information was connected and a smooth design has been created in order to facilitate the navigation of users through the platform and encourage them to return.

### Education Layer

**Figure 3:**
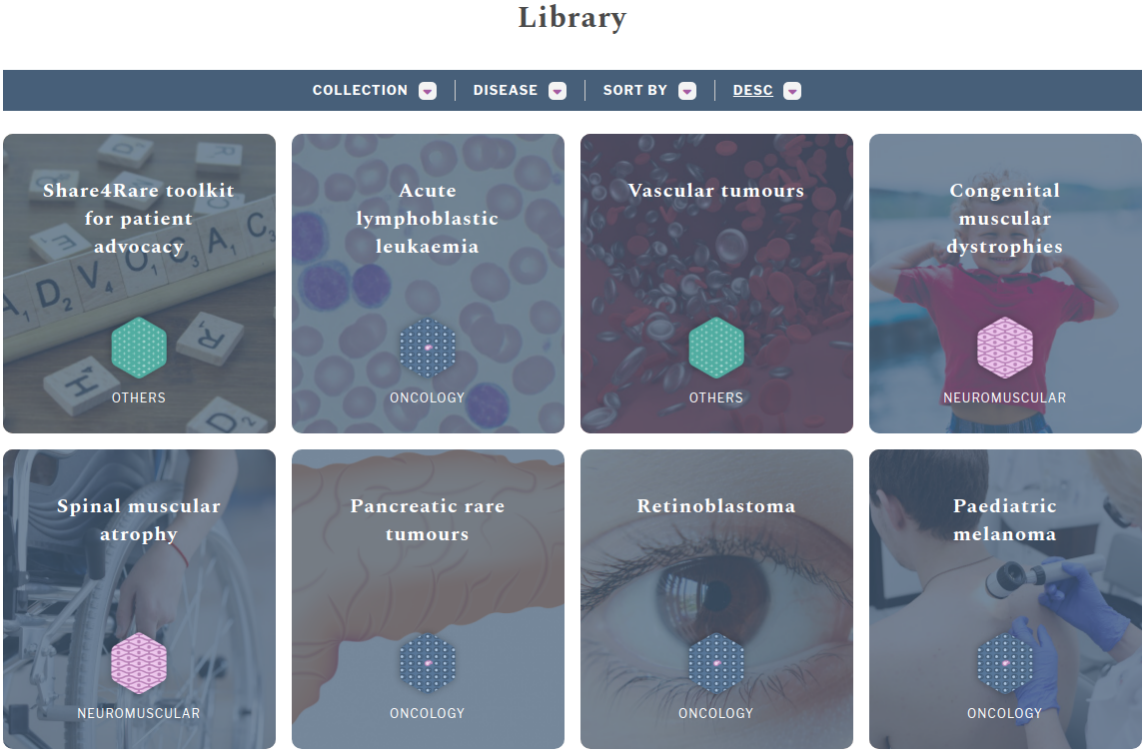
Screenshot showing some of the public medical materials available on Share4Rare.

The education layer will be a source of medical content for patients and caregivers within the rare disease community. During the platform development period, the platform will pilot over 3 groups of diseases (pediatric rare tumors, neuromuscular diseases, and undiagnosed patients), content for some of these diseases will be presented in the platform and offered to users. This material, as well as blogs and toolkits, will be publicly available (Figure 3).

### Reciprocity and Digital Karma

The basic functions of the community and research layers are based on the principles of reciprocity and digital karma, whose goals are to promote active and voluntary participation on the part of the users and to reward them for it.

The principle of reciprocity governs the relationship between each user and the rest of the platform and is bidirectional. In one direction, all contributions will always entail a reward (for instance, if a user completes a questionnaire, the user will gain access to enriched information related to it). In the reverse direction, to gain a benefit from the platform it is necessary to actively contribute to it (for instance, researchers who wants to register a questionnaire for their research must commit to being available to communicate actively with the rest of the users).

The principle of digital karma governs the profile of each user and their level of access to the functionalities that the platform implements. Essentially, the profile of each of the users of the platform is determined by their actions within Share4Rare: a user who contributes more information or who is more active in the community will see that this behavior is reflected on their profile and will be able to benefit from more functionalities.

### The Core of the Research Layer: Questionnaires

Medical and psychosocial questionnaires are central to Share4Rare. It is through them that the project will gather data, ensure the engagement of users and, most importantly, be able to generate new knowledge about rare diseases. The questionnaires will be designed by clinicians and other experts with expertise in the diseases covered by Share4Rare and will be web-based (on the platform). A subset of the answers given will be analyzed using statistical methods in an external server with the help of tools developed by a team of data scientists.

It is extremely important to obtain accurate and reliable information. Because of this, questionnaires use validation mechanisms for what is required, allowed values, minimum or maximum values and allowed formats for each question. Some of the questionnaires will be specific to a disease, while others may be applicable to several diseases or related to specific symptomatology.

Questionnaires will be provided to users (either patients or caregivers) progressively, so they can have control over which information they are sharing at every step and can decide whether to share it or not. As users advance through the questionnaires, they will have increasing feedback from the platform: (1) Each completed questionnaire will allow access to up-to-date comparative information about the answers that the rest of the community has given to the questionnaire and other topics related to it. (2) Each completed questionnaire will disclose more functionalities for the user (for instance, by giving them access to ask questions to the community or to open private messages with other users), and (3)The relevant data from each questionnaire will be incorporated to the user’s profile, which will increase their karma level and will allow the platform to better characterize the user in order to be able to present information of greater relevance.

All tools that the platform uses to collect data from the users (eg, registration forms, medical questionnaires, etc) included in the private environment of the platform require access using a user and password. The overall data collection and custody process of the platform is described in the Data management plan, a mandatory document in all Horizon 2020 European projects that is based the General Data Protection Regulation (mandatory and common across all European countries).

The Share4Rare platform and every pilot research project that will be performed until the end of the European Commission Grant have the approval of the Ethics Committee of *Sant Joan de Déu* Research Foundation. It is mandatory for all users of the platform to sign the informed consent document that regulates the use of clinical data and medical information for research purposes. If any use of these data occur in the future, the platform will ask for the re-consent of users that will be affected by this new use. Every year an external audit will be performed to oversee the use, gathering, and security of the data in Share4Rare.

### Conclusions

1. The platform aims to gather meaningful data from the patients who participate. Once duly treated and stored to guarantee patient anonymity and to ensure its statistical and clinical validity, said data will become part of a highly valuable repository that will be available for scientists and researchers conducting investigations in the field of rare diseases.

2. Share4Rare will develop a procedural framework that includes a set of validated tools that will allow researchers to gather much-needed data in a standardized manner, preserving its structural integrity, allowing for cross-comparison and cross-reference between studies and promoting the engagement of patients.

In parallel, the solutions that Share4Rare has devised to implement these 2 goals in the platform will allow clinicians and researchers to create a patient registry and will allow patients to be part of a community that makes it easier to connect with other people in their situation. At the same time, the platform will supply its participants with dynamic state-of-the-art content about the conditions, research being conducted, and other topics of interest to encourage active user involvement.
